# Outbreak of COVID-19 among children and young adults in a cancer centre daycare unit

**DOI:** 10.1017/S0950268822000012

**Published:** 2022-02-21

**Authors:** Ki Wook Yun, Ye Kyung Kim, Eun Sun Song, Hong Yul An, Kyung Taek Hong, Jung Yoon Choi, Hyoung Jin Kang, Seung Min Chung, In Kyung Park, Hyo Yeon Lee, Nam Joong Kim, Eun Hwa Choi

**Affiliations:** 1 Department of Pediatrics, Seoul National University College of Medicine, Seoul, Republic of Korea; 2 Department of Pediatrics, Seoul National University Children's Hospital, Seoul, Republic of Korea; 3 Seoul National University Cancer Research Institute, Seoul, Republic of Korea; 4 Wide River Institute of Immunology, Hongcheon, Republic of Korea; 5 Infection Prevention and Control Center, Seoul National University Hospital, Seoul, Republic of Korea; 6 Department of Internal Medicine, Seoul National University Hospital, Seoul, Republic of Korea

**Keywords:** COVID-19, SARS-CoV-2, nosocomial, children, immunocompromised

## Abstract

Nosocomial transmission of COVID-19 among immunocompromised hosts can have a serious impact on COVID-19 severity, underlying disease progression and SARS-CoV-2 transmission to other patients and healthcare workers within hospitals. We experienced a nosocomial outbreak of COVID-19 in the setting of a daycare unit for paediatric and young adult cancer patients. Between 9 and 18 November 2020, 473 individuals (181 patients, 247 caregivers/siblings and 45 staff members) were exposed to the index case, who was a nursing staff. Among them, three patients and four caregivers were infected. Two 5-year-old cancer patients with COVID-19 were not severely ill, but a 25-year-old cancer patient showed prolonged shedding of SARS-CoV-2 RNA for at least 12 weeks, which probably infected his mother at home approximately 7–8 weeks after the initial diagnosis. Except for this case, no secondary transmission was observed from the confirmed cases in either the hospital or the community. To conclude, in the day care setting of immunocompromised children and young adults, the rate of in-hospital transmission of SARS-CoV-2 was 1.6% when applying the stringent policy of infection prevention and control, including universal mask application and rapid and extensive contact investigation. Severely immunocompromised children/young adults with COVID-19 would have to be carefully managed after the mandatory isolation period while keeping the possibility of prolonged shedding of live virus in mind.

Although the proportion of children among the confirmed COVID-19 cases has increased with a relatively high vaccination rate in adults and the circulation of delta-variant severe acute respiratory syndrome coronavirus 2 (SARS-CoV-2) worldwide, children generally have an uncomplicated clinical course of COVID-19 [1]. However, high-risk children, including immunocompromised patients, could have more severe disease when infected with SARS-CoV-2 [2]. Here, we describe COVID-19 nosocomial outbreak control in the unique setting of a cancer centre daycare unit (CCDU) in a children's hospital. This study was conducted with approval by the Institutional Review Board (IRB) at Seoul National University Hospital (SNUH), and written consent was waived (IRB No. 2004-013-1115).

In November 2020, South Korea was continuing the strategy of stringent social distancing with universal face mask wearing in the community. Persons who were confirmed to have COVID-19 and those who were in close contact with confirmed cases were isolated in a designated hospital/facility and quarantined at home, respectively, for 14 days [3]. In the hospital setting, the entrance of the building was controlled, and all patients and visitors had to be checked for fever to be allowed to enter. Only one caregiver was allowed to stay with the patient. Everyone except young infants and patients with respiratory difficulty had to wear a face mask at all times during the hospital stay. This strategy was enforced with a penalty by the Seoul city local government since 13 November. Patients hospitalised for any reason had to have a negative test result for SARS-CoV-2 within the previous 72 h. These screening strategies were exclusively applied to hospitalised patients, except for those with half-day admission to the CCDU.

Seoul National University Children's Hospital (SNUCH) is a 1761-bed referral children's hospital in the capital city, Seoul, Republic of Korea. CCDU is a specialised admission unit for cancer patients to receive intravenous and/or intrathecal chemotherapeutic drugs as well as fluid therapy, transfusion and other injectable drugs for several hours. Patients are usually admitted in the morning, receive parenteral therapy and are then discharged within work hours on the same day. The beds inside the centre are located 1 m apart and separated just by curtain from each other. Caregivers usually stayed at the bedside while their children were receiving parenteral drugs (Supplementary Fig. S1).

On 18 November, an immunocompromised 5-year-old boy (patient A) visited the paediatric emergency department because of high fever in the early morning and was confirmed to have COVID-19. He was a patient with acute lymphoblastic leukaemia (ALL) and was admitted to the CCDU on 11 November. He was directly transferred to the designated isolation room for COVID-19 in SUNCH. In the meantime, we obtained information for another 5-year-old female cancer patient (patient B) and her mother (40 years old; caregiver B), who had visited CCDU together 2 and 5 days ago, and they tested positive for SARS-CoV-2 in an outside local hospital. They were tested on 18 November because of the mother's 1-day fever, but the patient was asymptomatic. They were also isolated to our hospital together on the following day (Supplementary Fig. S2).

These two epidemiologically linked cases, which had visited our CCDU within the past week, alerted the investigation for staff members in the CCDU. All staff members who had worked in the CCDU on the visiting days by confirmed cases were tested by PCR for SARS-CoV-2 and interviewed about suspicious contact and related symptoms. Then, a third COVID-19 case was confirmed in a 50-year-old female head nursing staff member on 18 November. On investigation, she had developed rhinorrhoea, cough and myalgia on 9 November and visited an outpatient clinic in the Family Medicine Department the next day. At that visit, she was not tested for COVID-19 and just received some medications for respiratory symptoms. She continued working at the CCDU until the day of COVID-19 confirmation. She usually received all the patients and caregivers first at the reception desk, registered the patient's admission to the unit and guided the patient to the bed inside (Supplementary Fig. S1). She sometimes performed simple venous line manipulation and/or blood sampling at a side desk and helped other staff members perform procedures, such as lumbar puncture. She wore a surgical mask whenever working, as the government and our institutional authority recommended. Where she contracted the virus from was not identified. We decided to regard her as the index case of this outbreak and initiated contact investigation for all patients, caregivers and staff visiting the CCDU between 9 and 18 November.

We listed a total of 473 exposures to the index case, which consisted of 181 patients, 242 caregivers, five co-visiting siblings and 45 staff members, based on the patients' visit log, work records of staff members and any other available documents. All listed persons were immediately informed about the exposure and recommended for PCR testing for SARS-CoV-2 anywhere available as soon as possible. A detailed contact investigation for defining high-risk contact was conducted just by personal/telephone interviews with confirmed and exposed persons because there was no video monitoring system in the CCDU.

Exposures were categorised at three levels: E1 for people who were family members of any confirmed COVID-19 case in this outbreak or who had close contact with the index case under improper mask wearing, E2 for close contact under proper mask wearing, and E3 for non-close contact with the index case. Close contact was defined as face-to-face contact for >15 min of continuous time or direct physical contact with the index. E1 persons were quarantined at home for 14 days and should have received a retest on the day before release. Both E2 and E3 persons were not quarantined if they had an initial negative COVID-19 test and were monitored for any symptoms actively by local government health personnel and passively by themselves, respectively, for 14 days after the last exposure. If there were new or worsening symptoms, testing was repeated. We recommended that any CCDU visit by E2 and E3 patients, if scheduled, be delayed for more than 2 weeks; otherwise, we recommended that they be retested for SARS-CoV-2 before the visit. For HCWs, E2 persons were initially excluded from work, retested 3 days later and then returned back to work with a negative result. Investigation for the community contact of confirmed cases was conducted by the local health authority.

In SNUH, SARS-CoV-2 was detected by a STANDARD™ M nCoV Real-Time Detection kit (SD Biosensor, Suwon, South Korea) targeting two separate genes, E and RdRp, via reverse transcription-polymerase chain reaction (RT-PCR). Whole-genome sequencing (WGS) of the selected SARS-CoV-2 was conducted using a NextSeq 550 Sequencing System. The results of contact investigation and SARS-CoV-2 tests were provided by the Infection Prevention and Control Center in SNUH. We retrieved the results of the investigation for the community contact of confirmed cases from the government's daily report of COVID-19.

As a result, a total of eight laboratory-confirmed COVID-19 cases, including the index case, were identified from 18 to 20 November, and no additional cases developed thereafter from this outbreak. Among 181 patients (median age of 8.0 years (range, 2 months to 29.4 years) and 42.5% female) who visited the CCDU from 9 to 18 November, 179 patients received at least one COVID-19 test (63.7% in SNUH and others in a local health authority/hospital) within 7 days after the beginning of the investigation, and three of them had a positive result (1.7%). In addition to the initially confirmed patients A and B, a 25-year-old male (patient C) was confirmed to be positive for COVID-19. The initial Ct values for the RNA-dependent RNA polymerase gene of SARS-CoV-2 were 15.3, 12.5 and 9.8 in patients A, B and C, respectively.

Three 1-year-old immunocompromised infants were categorised to E1 and quarantined at home for 14 days because their parents were confirmed to have COVID-19 (caregivers D–F). Another 7-year-old child was also disposed to self-quarantine because she had close contact with patient B without a proper mask. The other 174 patients, including two patients not receiving a SARS-CoV-2 test, were categorised as E2. None of them developed fever and/or respiratory symptoms during the monitoring period. Among the 242 caregivers (median age of 40.4 years (range, 22.2–73.0 years) and 71.1% female) and five siblings (median age of 5.4 years (range, 3.5–14.4 years) and 60.0% female) exposed (54.3% tested in SNUH), three mothers (40, 39 and 36 years old; caregivers B, D and E, respectively) and one father (35 years old, caregiver F) were confirmed to have COVID-19 (1.6%). Only caregiver B was infected together with her child (patient B). The Ct values were not informed to us for all these confirmed cases in caregivers who had been tested at outside facilities. No staff member except the index case patient was confirmed to have COVID-19 among 45 HCWs tested in SNUH (median age of 38.2 years (range, 18.9–65.0 years) and 75.6% female). The demographic characteristics of exposed persons and the results of contact investigations are summarised in Table 1. All confirmed cases were asymptomatic or mildly symptomatic and were referred to a World Health Organization severity score <4 (https://www.who.int/en/). There was no secondary case in our hospital or the community from these seven COVID-19 patients and caregivers during this outbreak.

**Table 1.** Demographic characteristics of exposed persons and results of contact investigation

	Patients	Caregivers/siblings	Healthcare workers
Number, *n* (%)	181 (38.3)	247 (52.2)	45 (9.5)
Age (year), median (range)	8.0 (0.2–29.4)	40.1 (3.5–73.0)	38.2 (18.9–65.0)
Female, *n* (%)	77 (42.5)	175 (70.9)	34 (75.6)
Characteristics, *n* (%)	Leukaemia, 50 (27.6)	Parent, 228 (92.3)	Doctor, 18 (40.0)
	Lymphoma, 14 (7.7)	Grandparent, 10 (4.1)	Nurse, 17 (37.8)
	Solid tumour, 85 (47.0)	Brother/sister, 6 (2.4)	Paramedic, 3 (6.7)
	Others^a^, 32 (17.7)	Others^b^, 3 (1.2)	Non-medical staff, 7 (15.5)
Last exposure, *n* (%)			
9–10 November	20 (11.0)	24 (9.7)	6 (13.3)
11–12 November	28^1^ (15.5)	34 (13.8)	5 (11.1)
13–14 November	30^1^ (16.6)	40^1^ (16.2)	4 (8.9)
15–16 November	43^1^ (23.8)	60^2^ (24.3)	11 (24.5)
17–18 November	60 (33.1)	89^1^ (36.0)	19 (42.2)
Contact investigation, *n* (%)			
Confirmed	3 (1.7)	4 (1.6)	0 (0)
Quarantined	4 (2.2)	3 (1.2)	11 (24.4)
Actively monitored	174 (96.1)	0 (0)	5 (11.1)
Passively monitored	0 (0)	240 (97.2)	29 (64.5)

a Langerhans cell histiocytosis (*n* = 10), haemophagocytic lymphohistiocytosis (*n* = 4), myelodysplastic syndrome (*n* = 4), aplastic anaemia (*n* = 8), chronic granulomatous disease (*n* = 1), Crohn's disease (*n* = 1), Gaucher disease (*n* = 1), mucopolysaccharidosis (*n* = 1), IgA deficiency (*n* = 1), systemic sclerosis (*n* = 1).

b Relative (*n* = 2) and guardian in the institute (*n* = 1).

The exposure, symptom development and isolation of confirmed persons are summarised in Supplementary Figure S2. Because patient A was receiving maintenance anticancer chemotherapy for ALL and patient B was monitored monthly after metaiodobenzylguanidine therapy for medulloblastoma, these two patients might not be severely immunosuppressed at the time of COVID-19 diagnosis, so they had an uneventful clinical course. However, patient C showed a unique clinical course and prolonged viral shedding. He received a second autologous peripheral blood stem cell transplant in October 2019 due to relapsed acute myeloid leukaemia. He received anti-CD20 therapy (rituximab) in February 2020 for Evans syndrome and had continued other immunosuppressive drugs (steroid and mycophenolate mofetil) for chronic graft-*vs.*-host disease (cGVHD) since then. He visited the CCDU daily to receive ganciclovir therapy for cytomegalovirus (CMV) reactivation during 9–13 November. He was diagnosed with COVID-19 on 18 November and admitted to the hospital the next day. He did not develop any new symptoms during CCDU visits or before COVID-19 diagnosis. Initial blood tests were normal except for preexisting anaemia and thrombocytopenia, and chest radiographic findings were also normal. Nasal congestion developed as the initial symptom of COVID-19 on 19 November and lasted for 3 days. During 22 November–11 December, he developed a fever, but there was no viral, bacterial or fungal infection or a flare-up of CMV during the COVID-19 illness. He was isolated for a total of 27 days due to the positive PCR test results for SARS-CoV-2 and prolonged fever without localised signs. Upon discharge from the hospital, patient C almost entirely stayed at home with only his mother, who had been tested for SARS-CoV-2 weekly and became diagnosed with COVID-19 on 9 January. The exact match of the WGS between patient C and his mother at the time when his mother became infected with SARS-CoV-2 highly suggests that this virus was transmitted from patient C to his mother. The Ct value on biweekly (during hospitalisation) or weekly (after discharge) RT-PCR tests for SARS-CoV-2 from patient C continuously decreased since the initial detection, and he finally tested negative on 15 February, which was 88 days after the COVID-19 diagnosis.

In this study, we demonstrated a COVID-19 outbreak from one staff member in the setting of a daycare unit for cancer patients in a children's hospital. The infection rate was 1.6%, and secondary cases did not develop during the outbreak. Nosocomial transmission of COVID-19 has the potential to induce high mortality in patients with underlying chronic disease and/or to spark community transmission [4]. Prevention and control of nosocomial infection might be more difficult with COVID-19, as viral shedding from asymptomatic cases might play a major role than with other infectious diseases. In a report of nosocomial COVID-19 transmission over one month in a Japanese hospital, 95.5% of transmission events were suspected of having occurred during the asymptomatic (mostly presymptomatic) period of patients [5]. However, the infection rates of HCWs were very low with the proper use of personal protective equipment (PPE) with masks when a nosocomial outbreak of COVID-19 developed. Moreover, large-scale contact investigation and isolation contributed to the termination of nosocomial infection and prevention of community transmission of COVID-19 [5, 6].

In the current study, diagnosis of the initial three nosocomial COVID-19 cases led to rapid contact tracing, symptom screening, extensive testing and limited quarantine of exposed persons. Among the eight confirmed cases, including the index case, six were symptomatic, and two were asymptomatic during their whole investigated and quarantined periods. Half of the symptomatic cases had mild respiratory and/or systemic symptoms for only 1 day. These paucisymptomatic illnesses might not give attention to the possibility of in-hospital transmission of SARS-CoV-2. Therefore, the baseline IPC strategy would be important for COVID-19 prevention and control. In our hospital, universal mask application inside the building became mandatory for all HCWs as well as patients, caregivers and visitors during the period of this outbreak. Partly due to all these efforts and policies, the outbreak size was relatively small despite the exposure time of 9 days from the index case, and we were able to successfully control the outbreak without any in-hospital secondary cases. In addition, although all immunocompromised patients on care by our CCDU and their caregivers followed the same IPC rules for COVID-19 in both community and hospital, they were probably more stick to rules than the other healthy persons in the community.

There are very limited reports on the COVID-19 outbreak among immunocompromised children in the hospital setting. In a nosocomial outbreak of COVID-19 beginning with a previously healthy 9-year-old girl in a paediatric ward in a tertiary care hospital in South Korea, 1152 contacts underwent SARS-CoV-2 tests [7]. The results were negative for all of the close and casual contacts, including the index patient's parents and a total of 18 patients and caregivers staying in the six-patient room with the index patient except for one mother of another infant who shared the six-patient room directly across from the index patient (an approximately 3 m distance). Additionally, in the current study, there were no secondary COVID-19 cases from two confirmed children in the hospital and the community, even though they were probably isolated 2–7 days after exposure.

Infectious SARS-CoV-2 is generally not detected after 7 days or more after symptom onset, and contact tracing studies suggest that individuals are most infectious within 5 days of symptoms [8]. Because RT-PCR positivity appears to persist for several weeks, national guidelines have suggested that viral RNA detected after 2 weeks of illness is of unclear significance and favour basing isolation decisions on time since symptom onset (www.cdc.gov). However, several recent reports on the prolonged shedding of infectious SARS-CoV-2 in immunocompromised patients were published from the United States [9, 10]. In the current study, patient C had profound immunosuppression for cGVHD after undergoing HSCT and B-cell immunosuppression after receiving cellular therapies (anti-CD20) for Evans syndrome. Although our patient had shown relatively mild symptoms and a clinical course compared with those in previous studies [9, 10], he shed SARS-CoV-2 for at least 12 weeks and transmitted the virus to his mother approximately 7–8 weeks after the initial infection. Severely immunocompromised patients would have to be managed more cautiously because they could transmit the live virus during the post-symptomatic period.

This study has several limitations. We could not identify the SARS-CoV-2 infection source of the index case patient; therefore, we could not confirm that the index case was the primary case in this outbreak. However, it was highly suspicious because her symptoms developed much earlier than those of any other confirmed cases. Although a rigorous contact investigation of every confirmed case could not find any other infection source of SARS-CoV-2 in the community, even relatively with low infection pressure and no domestic variant virus detected at that time (0.6 daily confirmed nationwide COVID-19 cases per 100 000 population on 18 November 2020; http://ncov.mohw.go.kr/), we could not totally exclude the possibility of community-acquired COVID-19 among our cases. Because we performed the detailed contact investigation only by interviews, the actual transmission route and behavioural risk factor for the confirmed cases could not be investigated. Additionally, the possibilities of asymptomatic, not-tested secondary COVID-19 cases in the community and community-transmitted cases among the seven cases in this outbreak were present even though the national investigation for community contacts was very stringent in South Korea. Last, we did not perform SARS-CoV-2 viral culture in patient C to confirm the infectivity or WGS for the contemporary SARS-CoV-2 in the community to exclude another source of the virus transmitted to his mother. This might limit the confirmation of the prolonged shedding of live virus from patient C.

In conclusion, the nosocomial outbreak of SARS-CoV-2 can be limited with proper PPE with universal mask wearing of all persons staying in the hospital and with the immediate isolation and thorough PCR testing of patients and HCWs, together with extensive contact tracing and social distancing measures when an outbreak is suspected. Immunocompromised children and young adults may also have mild symptoms and an uncomplicated clinical course but may become persistent shedders and sources of SARS-CoV-2 transmission, particularly when they have profound immunodeficiency. Therefore, these cases should be carefully managed.

## Data Availability

The corresponding author had full access to all the data in the study.

## Supplementary material

For supplementary material accompanying this paper visit https://doi.org/10.1017/S0950268822000012. click here to view supplementary material
