# Supplementary material for: Outbreak of COVID-19 among children and young adults in a cancer centre daycare unit
Source: Epidemiol Infect. 2022 Feb 21;150:e40. doi: 10.1017/S0950268822000012 (PMC8886074; doi:10.1017/S0950268822000012)
Supplement: Supplementary file 1 [file hygsup.zip › S0950268822000012sup002.pptx]

## Slide 1
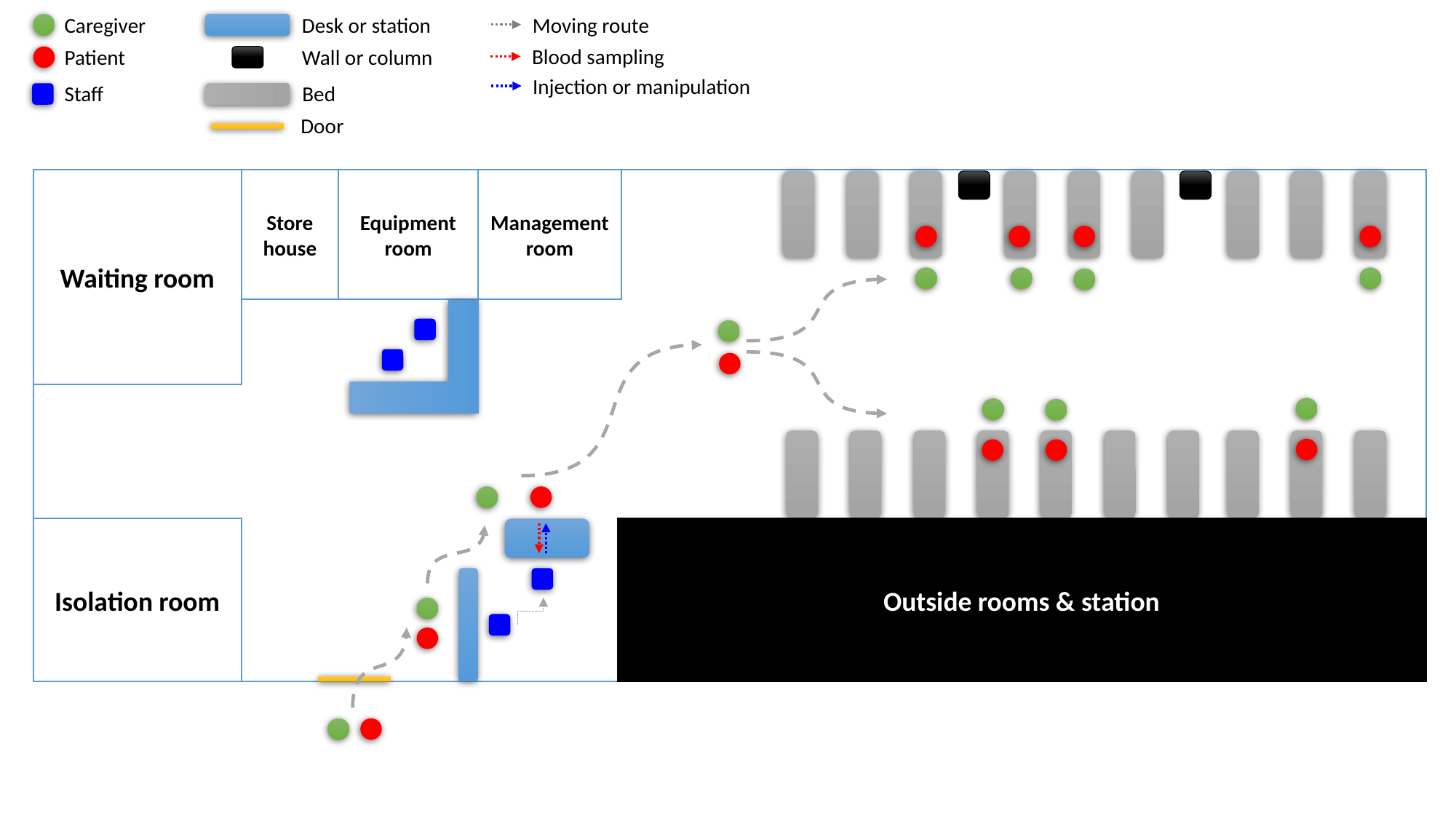

Caregiver
Desk or station
Moving route
Blood sampling
Wall or column
Patient
Injection or manipulation
Bed
Staff
Door
Management room
Waiting room
Store house
Equipment room
Isolation room
Outside rooms & station
