# Supplementary figures and images for: Outbreak of COVID-19 among children and young adults in a cancer centre daycare unit
Source: Epidemiol Infect. 2022 Feb 21;150:e40. doi: 10.1017/S0950268822000012 (PMC8886074; doi:10.1017/S0950268822000012)

## Slide 1
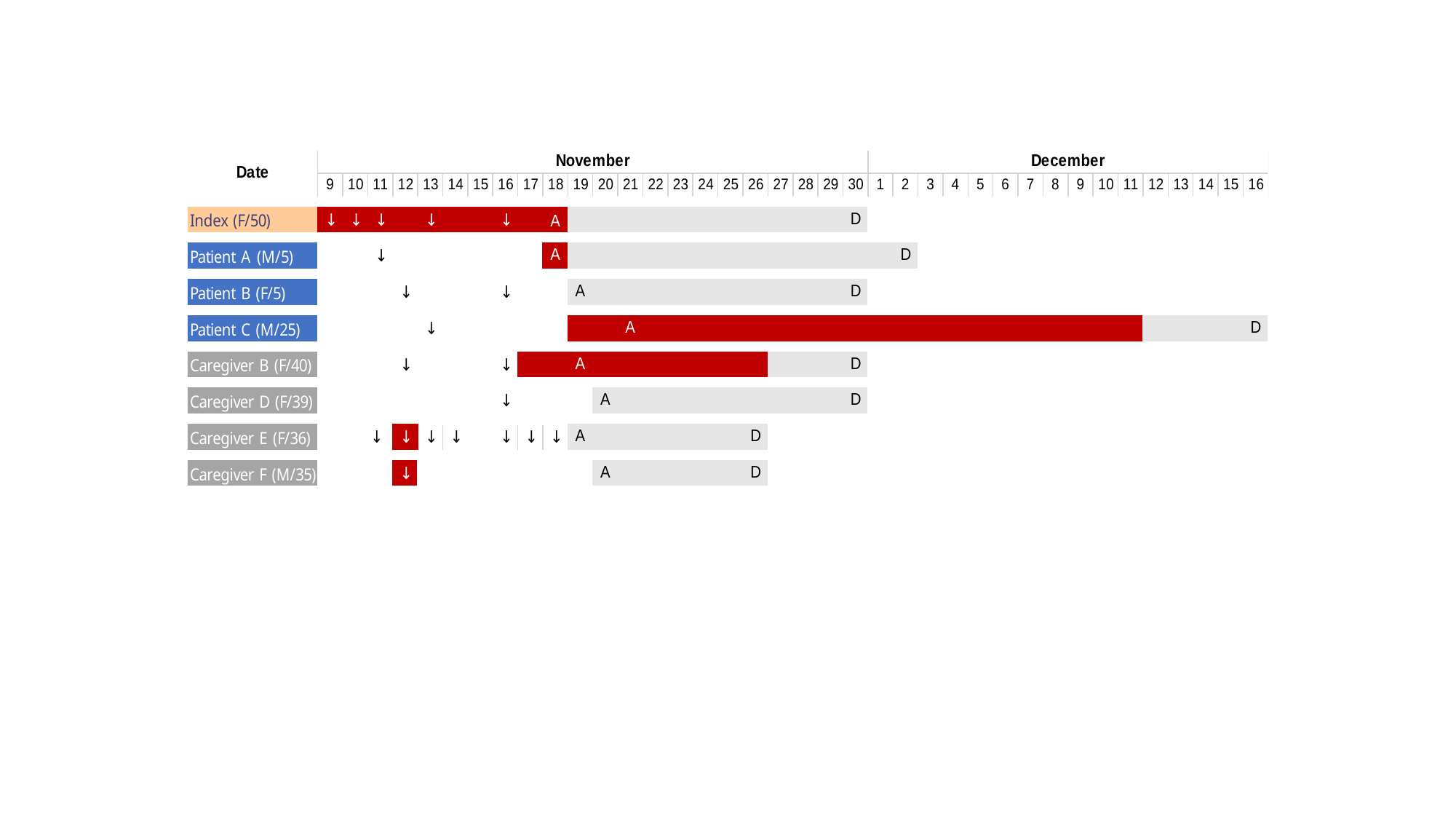

Supplement: Supplementary file 1 [file hygsup.zip › S0950268822000012sup001.pptx]
